# Death Anxiety Associated With Coronavirus (COVID-19) Disease: A Systematic Review and Meta-Analysis

**DOI:** 10.1177/00302228211050503

**Published:** 2021-10-08

**Authors:** Safiye Özgüç, Emine Kaplan Serin, Derya Tanriverdi

**Affiliations:** 1 Department of Psychiatric Nursing, The Faculty of Health Sciences, Gaziantep University, Gaziantep, Turkey; 2 The Faculty of Health Sciences, Gaziantep University, Gaziantep, Turkey

**Keywords:** COVID-19, death anxiety, mental health, prevention, systematic review, meta-analysis

## Abstract

Death anxiety is a feeling that exists since birth, continues throughout life, lies at the root of all fears, and develops after the awareness that people will no longer exist and that they can lose themselves and the world. It is associated with death-induced anxiety with many features of the COVID-19 pandemic, which can be seen as a threat to human existence. In this study, it is aimed to compile studies on death anxiety associated with coronavirus disease, list the general characteristics of these studies through descriptive summaries, and combine findings on the level of death anxiety associated with coronavirus disease through meta-analysis. The findings of this study suggest that COVID-19 pandemic process and is affected by socio-demographic factors such as fear of COVID-19, gender, and occupation. As a result of this study, it was determined that death anxiety was relatively high during the COVID-19 pandemic process.

Coronavirus (COVID-19), which affects the whole world, is a virus that manifests itself with acute respiratory syndrome in humans and has turned into a pandemic. Because pandemics threaten many people with the same disease, they affect societies differently from classical diseases. Pandemics cause large masses to live in fear and anxiety and disrupt the natural flow of life. The constant spread of the disease, high morbidity and mortality cause a common fear and anxiety and negatively affect the mental health of individuals. In addition, pandemics can cause negative effects on mental health by reminding people all over the world of the fact of death (Bostan et al., 2020; Çağlar & Kaçer, 2021; Okuyan et al., 2020). When the psychological effects of the COVID-19 pandemic were evaluated in terms of social mental health, it was found that people experienced the highest levels of stress or anxiety (WHO, 2020).

As of the beginning of the study, the total number of patients related to COVID-19 worldwide was reported as 2.220.855 and the total number of deaths was reported as 21.093. When the study was completed, the number of patients infected with COVID-19 worldwide was 4.820.591 and the total number of deaths was 40.131 (https://www.worldometers.info/coronavirus/). One of the types of death that affects societies in sociological and psychological terms is mass deaths during crisis periods. Since the coronavirus pandemic process causes the death of thousands of people, it can reveal death anxiety in individuals or exacerbate the existing anxiety (Turhan, 2021). Death is constantly remembered due to the ever-increasing number of deaths updated daily from news about COVID-19 and social media, and the encounter with protection methods such as face masks, antibacterial sprays and handkerchiefs that remind the disease everywhere (Menzies & Menzies, 2020; Menzies et al., 2020). In addition, debates such as the end of the world, panic, death, and the number of cases in the media also remind about death (Özyürek & Atalay, 2020). With the quarantine applied to protect against pandemic, the situation of staying at home continuously, the decrease in social relations and the feelings of loneliness, the unexpected deaths of loved ones, the presence of many images reminding of death, frequent and repeated washing of the hands, and disturbing thoughts about the disease negatively affect mental health (Damirchi et al., 2020; Özyürek & Atalay, 2020). These factors cause psychological problems in individuals such as depression, fear (especially fear of death), anxiety of not getting adequate and efficient health care, sleep problems, anxiety (especially death anxiety) (Okuyan et al., 2020). It can be said that death anxiety has increased especially due to the events experienced in the COVID-19 pandemic (Menzies & Menzies, 2020; Menzies et al., 2020).

Fear of death or death anxiety is present in all humans but becomes more pronounced in important situations related to death (Menzies & Menzies, 2020). In terms of contemporary existentialist thinking, “fear of death” or “death anxiety” is the most fundamental problem of man (Kandemir, 2020) and death anxiety can decrease individual well-being (Robah, 2017). It has been suggested for over a hundred years that death anxiety is at the core of being human and certain fears (Menzies & Menzies, 2020; Menzies et al., 2020). It has been suggested that death anxiety has a transdiagnostic nature that underlies number of different mental health conditions. For example, fear of death can be seen as frequent seeking reassurance from physicians through self-control and continuous analysis of somatic symptom-related disorders (Menzies & Menzies, 2020). In addition, the increase in stress causes excessive density in health institutions as well as physical health problems (Biçer et al., 2020). For this reason, people use many defense mechanisms to deal with these situations.

Studies show that people are in tension and anxiety during the COVID-19 pandemic. In this case, effective use of mindfulness and coping strategies will help people control stressful events and reduce negative emotions (Damirchi et al., 2020). Therefore, death anxiety should not be ignored during the COVID-19 pandemic.

In this study, it is aimed to (a) compile studies on death anxiety associated with coronavirus disease, (b) list the general characteristics of these studies through descriptive summaries, and (c) combine findings on the level of death anxiety associated with coronavirus disease through meta-analysis. Thus, it is aimed to make a judgment about the death anxiety experienced in this process. The results of this study are intended to provide data that will assist future preventive and therapeutic studies on death anxiety associated with coronavirus disease.

## Methods

This systematic review and meta-analysis was conducted in accordance with the Preferred Reporting in Systematic Reviews and Meta-Analyzes (PRISMA) guideline (http://www.prisma-statement.org/documents/PRISMA%202009%20checklist.pdf). This meta-analysis was prospectively registered in PROSPERO (International Prospective Register of Systematic Review) (registration number: 243873).

To access COVID-19 Related Death Anxiety research, Google Academic, ScienceDirect, Academic Search Complete (EBSCOHOST), EMBASE, MEDLINE, CINAHL, Web of Science, Cochrane and Pubmed databases were searched in 01 January-30 April 2021. For the selection of keywords, medical subject heading (MesH) terms were used based on PICO (Population Intervention, Compare, Outcome). While scanning the literature, the keywords “Coronavirus”, “ COVID-19”, “Pandemic”, “Death anxiety” and “Death distress”, “Fear of death” were used in English and Turkish.

**Inclusion criteria in the review study:** Studies involving death anxiety associated with coronavirus (COVID-19) disease, descriptive and quasi-experimental research design, accessible online full text, original and quantitative studies published in a refereed journal in 2020–2021 in Turkish or English, and the full text has been reached were included in the review study.

**Exclusion criteria in the review study:** Studies outside the scope of Death Anxiety Associated with Coronavirus (COVID-19) disease have been identified as case reports, panel presentations, reviews, unpublished theses, and ongoing studies were excluded from the review study.

**Figure 1.**
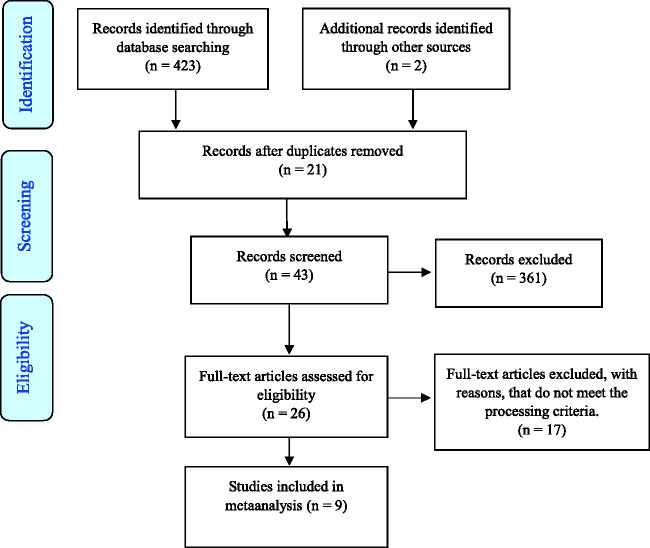
Study Flow Diagram.

As a result of the keyword and literature search, a total of 425 studies were reached. The accessed 425 studies were primarily examined according to the titles and 361 studies that were not related to the research subject were excluded. The abstracts and full texts of 43 studies were evaluated in terms of inclusion and exclusion criteria, and 26 studies were reached in accordance with the inclusion and exclusion criteria. These 26 studies were included in the systematic review section of the study. However, four of the 17 studies were excluded from the scope of meta-analysis because they did not contain the necessary information. Nine of these studies were included in the meta-analysis (Figure 1).

### Data Collection

Three researchers independently made a literature review in the study. Summaries of potential studies and conflicts were resolved by consensus. Full text articles of potential studies were independently read by the researchers to identify studies that met the inclusion criteria. Reference lists of all included studies and reviews have been reviewed for eligible articles.

### Critical Appraisal

The methodological quality assessment of the studies included in the study was evaluated by at least two independent researchers using the “Meta-analysis of statistics assessment and review instrument (JBI-MAStARI)” developed by the Joanna Briggs Institute (JBI) according to the selected research types. For each item in the JBI-MAStARI checklists, a “Yes” answer is evaluated with 1 point, “No”, “Not specified” and “Not suitable” answers are evaluated with 0 points. The Critical Assessment score ranges from 0 to 9, and the higher the total score, the higher the methodological quality of the research (https://jbi.global/critical-appraisal-tools; Nahçıvan & Seçginli, 2015). The quality of the studies is based on inclusion criteria, coding required meta-analysis data, and special indicators (such as death anxiety mean, sample size, psychometric data of measurement tools). The selection of the studies for meta-analysis was carried out by three researchers separately. As a result of the evaluation made by all three researchers, the highest score was 9 and the lowest score was 7. In the comparison made, it was determined that there was 100% agreement among the researchers on the inclusion criteria. The reliability of the coded data was provided by comparing the coding of the researchers. 100% agreement between coders was met.

### Measures

#### Templer's Death Anxiety Scale (DAS)

The Death Anxiety Scale developed by Templer has two forms: First; consists of 15 statements. Answers are Yes/No. One of the scale items is a ‘‘life after death worries me a lot’’. The scoring is one (yes) or zero (no). The lowest score that can be obtained from the scale is 0, and the highest score is 15. The cut-off point of the scale is 6–7 and above indicates very high death anxiety symptoms. Templer reported reliability for the total scale to be 0.83 (Templer, 1970). It has been reported that as the score obtained from the scale increases, death anxiety increases. Latter; It consists of 15 statements and is in five-point likert type. While the lowest score that can be obtained from the scale is 15, the highest score is 75. The scale scoring is 15–35 low, 36–55, medium and 56–75, high. It has been reported that as the score obtained from the scale increases, death anxiety increases. Cronbach’s alpha and split-half coefficients for DAS were reported as 0.76 and 0.87 respectively for a sample of older adults and college students (Conte et al., 1982; Templer, 1970)

#### Revised Death Anxiety Scale (R-DAS)

It was developed by Thorson and Powell. It consists of 25 items in total. Each item of the 5-point Likert type scale is scored as “0 = Strongly Disagree”, “1 = Disagree”, “2 = Neutral”, “3 = Agree” and “4 = Strongly Agree”. One of the scale items is a ‘‘the pain involved in dying frightens me’’. The lowest score that can be obtained from the scale is “0” and the highest score is “100”. High scores mean higher anxiety (Karaca & Yıldız, 2001). In the original study The Cronbach alpha of reliability calculated for these data was .804 (Thorson & Powell, 1992).

#### Arabic Scale of Death Anxiety (ASDA)

The Arabic Death Anxiety Scale (Abdel-Khalek et al., 2009), which was approved as a result of a research conducted in three Arab countries, is a five-point Likert structure containing 20 items. One of the scale items is a ‘‘I fear death’’. ASDA's total score ranges from 20 to 100. The higher the score obtained from the scale, the higher the level of death anxiety. In the original study, the ASDA had very satisfactory Cronbach’s alpha reliability scores ranging from 0.88 to 0.93, and good internal consistency scores ranging from 0.74 to 0.90 (Abdel-Khalek et al., 2009).

### Procedure

In the first part of the study, the characteristic features of the studies included in the analysis are given (Table 1). The analysis of the studies included the author, publication year, study type, sample number, age, and evaluation results of death anxiety. In the second part of the study, in the light of data such as arithmetic mean, standard deviation and sample size obtained from the studies, effect sizes method calculated from the types of meta-analysis of the means was used in analyzing the data. Comparison of the averages of each study and these were calculated using the statistical software CMA (The Comprehensive Meta-Analysis software) for meta-analysis.

**Table 1.** Characteristics of the Studies.

Author, Year	Country	Sample size	Sex % Female	Mean age	Death Anxiety Assessment	Results	Quality
1. Damirchi et al. (2020)	Iran	300	**%**50	**Mean Age (18–30)** Female: 78; Male: 81**Mean Age (31–54)** Female: 72; Male:69	TDAS(Yes/No)	As a result of the correlation analysis, it was found that there was a significant relationship between self-talk, death anxiety, obsessive-compulsive disorder and coping strategies. In addition, there is an inverse relationship of −0.41 and −0.243 between self-talk and death anxiety and obsessive-compulsive disorder. Self-talk can predict death anxiety, obsessive-compulsive disorder, and coping strategies (p <.001).	**9**
2. Gokdemir et al. (2020)	Türkiye	240	%58	Mean Age: 40.58 ± 8.88	TDAS(Yes/No)	There is a significant negative correlation between behavior, attitude, and death anxiety towards COVID-19: r (250) = −0.22, P < 0.01. Fear of COVID-19 was found to be associated only with death anxiety, r (250) = 0.27, P < 0.01. It is reported that fear of COVID-19 is higher in those with higher death anxiety.	**8**
3. Guner et al. (2021)	Turkey	354	% 23.4	**Mean Age**: 68.28 ± 2.9065 years and overFemale:83 Male: 271	TDAS(Yes/No)	As a result of the study, a significant difference was found between death anxiety and loneliness in the elderly.	**9**
4. Söğütlü & Göktaş (2021)	Turkey	789	%75.7	**Mean Age**: 21.51 ± 3.9718–26 yaş arasıFemale: 597 Male: 192	TDAS(Yes/No)	The death anxiety level of university students caused by the pandemic was found to be 48.3%. The person's history of COVID-19 contact, and hospital admission, or hospitalization in a first-degree relative and a positive test results make the effect of health anxiety on death anxiety and somatic symptom severity and the effect of death anxiety on somatic symptom severity meaningful.	**8**
5. Mirhosseini et al. (2021)	Iran	1215	%47.1	**Mean Age**: 33.70 ± 10.69 Female: 643Male: 572	TDAS(Yes/No)	49.1% of all participants included in the study reported high death anxiety. In parallel with the high anxiety level, a significant increase in death anxiety was observed (p < 0.001). In addition, factors such as younger age and the death of a family member from COVID-19 were found to be significantly associated with death anxiety (p = 0.024 and p = 0.001).	**9**
6. Shakil et al. (2021)	Pakistan	468	%46	Mean Age: 29.71 ± 11.15	TDAS (Beşli likert)	As a result of the correlation analysis, a moderately positive correlation was found between psychological distress and death anxiety (r = 0.343 ***, p <.001). It revealed a meaningful moderator role of work situation, showing the strong relationship between psychological distress and anxiety for the non-working participants. Therefore, psychological distress is more prominently associated with death anxiety in those who do not work than those who work.	**8**
7. Sanadgol et al. (2020)	Iran	50	%56	Intervention GroupMean Age: 33.56 ± 5.57Control Group Mean Age: 31.24 ± 5.21	TDAS (Beşli likert)	The mean death anxiety score shown after one month of GI (guided imagination) in the intervention group decreased from 53.28 to 43.48 (p = 0.01). There was no significant difference before and after the intervention in the control group (p > 0.05).	**7**
8. Kandemir (2020)	Türkiye	660	%51.7	Mean Age: 15–64	ÖKÖ	The level of death anxiety significantly differentiates according to the marital status of the participants (p <.01). While the death anxiety score of married individuals is (X = 46.4), it is (X = 50.6) for single participants. According to the findings, it can be said that singles have more death anxiety than married people.	**7**
9. Rababa et al. (2021)	United arabemirates	248	%42.3	Mean Age: 63.95 ± 2.9	ASDA	The study aimed to examine the relationship between death anxiety and religious coping and spiritual well-being among older adults in the COVID-19 pandemic. The majority of the older adults who participated were found to have low levels of religious coping and spiritual well-being and high levels of death anxiety. Moreover, compared to older male adults, female older adults were found to have higher levels of religious coping and lower levels of death anxiety. It was also found that married older adults have higher levels of death anxiety compared to widowed older adults. After controlling for sociodemographic characteristics, religious coping and mental well-being were found to be important predictors of death anxiety in older adults.	**8**
10. Pradhan et al. (2020)	İndia	200	%61.5	Mean Age: 24.64 ± 4.49	DAQ	It revealed significant gender-based differences in the religious coping and death anxiety levels of the participants. These are (t246 =,. 72, p < 0.001) and (t245 = −7.26, p < 0.001), respectively. Compared to male older adults, female older adults reported higher levels of religious coping and lower levels of death anxiety. In addition, a significant difference was found between the death anxiety levels of married older adults and widowed elderly adults. Married older adults had higher death anxiety.	6
11. Chen et al. (2020).	China	917	% 66.85	Mean Age: 28.6 ± 9.47	DAQ	The media had a positive relationship with empathy, sympathy, positive emotion, negative emotion, and death anxiety. Death anxiety was positively associated with empathy, sympathy, and negative emotion.	8
12. Yıldırım & Güler (2021)	Türkiye	3109	%51.02	Mean Age: 38.64 ± 10.40	DDS	The results show that perceived risk has a significant direct effect on positivity, death anxiety and happiness. Positivity has an important direct effect on death anxiety and happiness. Mediation analysis showed that positivity mediates the effect of perceived risk on death anxiety and happiness.	7
13. Kavaklı et al. (2020)	Türkiye	562	% 64.4	Mean Age: 33.53 ± 10.92	Turkish DAS	As a result of the study, the death anxiety levels of the female participants (Mean = 40.41, SD = 21.18) were significantly higher than the male participants (Mean = 26.32, SD = 19.52).	8
14. Lázaro-Pérez et al. (2020)	Spain	157	%79	Mean Age <41: 47.8	Collett–Lester Death Anxiety Scale	The study was conducted to determine whether healthcare workers in Spain produce anxiety about the death processes of their patients and which variables this anxiety covers. The Anxiety Related to Death Risk of the Patients Logistic regression model was found to be statistically significant, X2 = 24.100, p < 0.005. The model explains 22.4% of the variance (Nagelkerke's R2) in the risk of death anxiety versus deaths of others.	8
15. Lázaro-Pérez et al. (2020)	Spain	2079	%12.5	31–40 Mean Age:35.2	The Collett Lester Fear of Death Scale	The scale shows some higher levels of fear of death of others (82.1%) and fear of dying of others (78.2%) as well as 69.2% of the total. On the other hand, from binary logistic regressions, four variables that condition the risk of suffering from death anxiety have been proven: (a) certainty of need for future psychological treatment, (b) absence of individual protection equipment (PPE), (c) high level of Emotional Exhaustion, (d) high level of depersonalization. These latter two come from the Maslach and Jackson Burnout scale.	8
16. Zhang et al. (2020)	China	7678	%88.46	M: 34.88 ± 10.468	TDAS (Yes/No)	When the surveys applied one month after the pandemic started and during the pandemic process, the number of individuals with death anxiety increased from 48.1% to 53.2%. High death anxiety has also been identified as an important factor affecting PTSD. Compared to other professions, healthcare professionals have been found to have more persistent death anxiety during the COVID-19 pandemic.	8
17. Ring et al. (2020)	Israel	277 olderadults	%69	Mean Age:69.59 ± 6.72	DASC	This study showed that subjective proximity to death attenuates the association between health concerns and death anxiety among older adults during the pandemic. It has also been shown that older adults who report high levels of health concerns related to COVID-19 also report higher levels of death anxiety.	8
18. Çağlar & Kaçer (2021)	Turkey	360	%45	Mean Age: COVID-19 Pneumonia:57MI:59Control:45	RDAS	In a study conducted with 120 patients with myocardial infarction (MI), 120 patients with COVID-19 pneumonia and 120 healthy volunteers to determine the state / persistent and death anxiety levels of patients with myocardial infarction (MI) and COVID-19 pneumonia, all scores of the patient groups were found to be significantly higher than the control group. There was no significant difference between the COVID-19 pneumonia and MI groups in terms of STAI-S, STAI-T and RDAS scores. Patients with COVID-19 pneumonia increased anxiety risk by 2.14 times for STAI-T and 1.97 times for STAI-S compared to the control group.	8
19. Simione & Gnagnarella (2020)	Italy	353	%75.07	Mean Age: 38.26 ± 12.24Females = 265Males = 88	ECQ	As a result of the study, healthcare workers reported a higher risk perception and anxiety level regarding COVID-19 infection compared to the general population. It has been found that there is a difference in risk perception between healthcare workers and the general population.	7
20. Indacochea et al. (2021)	Latin America	219	%47.95	Mean Age: 49.90 ± 11.91Females = 105Males = 114	TDAS (Yes/No)	The study was conducted to evaluate the individual's perceptions and responses to mortality among Latin American physicians treating COVID-19 patients. Participants' fear of death ranges from 56.2% to 90%. Also, the prevalence of "High anxiety" was 80.8%. A statistically significant correlation was found between the fear of death of others according to age and time after graduation (p = 0.010 and p = 0.020, respectively). As a result, it has been stated that death anxiety is high for doctors treating COVID-19.	8
21. Kirthiga (2020)	India	112	%78	Male : %22Females = 87Males =25	DAS	It was stated that there is no significant difference between Indians and foreigners in terms of endurance and death anxiety. The study also showed that there was no significant difference in death anxiety in men and women.	8
22. Chodkiewicz et al. (2021)	Poland	618	%80.9	Mean Age: 26.04 ± 9.74	SDA	The study aimed to examine the relationship between mental health and death anxiety, fear of coronavirus, perceived stress level, alcohol consumption and coping strategies with pandemic stress during the second wave of the pandemic. As a result of the study, it was stated that those with both anxiety and depressive disorders were in the worst condition and they felt the fear of death and coronavirus the most.	7
23.Ring et al. (2020)	Israel	277	%69	Mean Age: 69.59 ± 6.72	FDS	The study was conducted to examine whether proximity to subjective death mitigates the association between health concerns and death anxiety due to the COVID-19 outbreak among older adults in Israel. It has been demonstrated that subjective proximity to death attenuates the relationship between health concerns and death anxiety. It was also noted that older adults who reported high levels of health concerns related to COVID-19 also reported higher levels of death anxiety.	8
24. Jagadeesan (2020)	India	231	%89.60	Mean Age:25.70	TDAS (True/False)	As a result of the regression analysis, it is revealed that among the seven variables (Body Vigilance, Disgust Sensitivity, Disgust Tendency, Prediction, Luck, Pessimism, and Death Anxiety), the most appropriate model includes only three variables: death anxiety, disgust sensitivity, and luck. Death anxiety emerged as the strongest predictor (ß = 4.76, p <.01)	8
25. Bentall et al. (2021)	UK	3066	–	–	DAI	As a result of the study conducted to test the psychological model of excessive buying and panic buying in the COVID-19 pandemic, only depression and death anxiety among the psychological distress variables were found to be significant.	8
26. Awosola & Aıkpoghomhe (2020).	Nijerya	315	%45.8	Mean Age:39	COVID-19 Death Anxiety Scale	The research is designed to develop the COVID-19 death anxiety scale, due to the anxiety and high mortality rate associated with the Coronavirus (COVID-19) outbreak. Psychometric properties of the COVID-19 Death Anxiety Scale showed that the scale is reliable and valid.	7

*Note.* TDAS = Templer Death Anxiety Scale; ASDA = Arabic Scale Of Death Anxiety; RDAS = Thorson–Powell’s Revised Death Anxiety Scale; ÖKÖ = Ölüm Kaygısı Ölçeği; DAQ = Death Anxiety Questionnaire ; DDS = Death Distress Scale; Turkish DAS = Turkish Death Anxiety Scale; DAS = Death Anxiety Subscale; STAI-T = State-Anxiety Inventory-Trait; STAI-S = State-TraitAnxiety Inventory-State; FDS = Fear of Death Scale; DAI = Death Anxiety Inventory; SDA = Scale of Death Anxiety; ECQ = The Death Anxiety Scale of the Existential Concerns Questionnaire.

### Ethical Consideration

Since the literature review model is used in the study, it does not have a direct effect on humans or animals. Therefore, there is no need for an ethics committee approval decision.

### Data Analysis

For the analysis, firstly, the effect sizes of the studies included in the research were calculated. Effect size is considered as the basis of meta-analysis studies. One variable relationship is the examination of values such as mean, median, mode. In other words, it is a value that reflects the size of the application effect. In cases where the result is reported with a meaningful scale and the same scale is used for all studies in the analysis, the raw differences in direct means can be used as effect size. When combining the results of the research in meta-analysis and calculating the pooled estimate, either the fixed effect model or the random effect model is used. These two models estimate the average effect value by weighting the studies differently. The fixed effect model is based on the assumption that all studies collected in the context of meta-analysis share similar effect sizes. The purpose here is not to predict the effect size, but to estimate the mean of the distributions. Fixed effect models assume that the variance between study results arises from data related to each other. This model can explain the variability other than sampling error with other variables in meta-analysis. These variables can systematically differentiate studies with large or small effect sizes. In cases where all studies are functionally the same and it is desired to calculate the pervasive effect for the population in which the studies are conducted, it is more appropriate to use a fixed effect model. In cases where homogeneity is met in meta-analysis method, “fixed effect” model is used. In addition, if working with a fixed effect, meta-analysis can be applied with both studies. In this study, both the fixed effect model and the random effect model were used (Biçer et al., 2020; Dinçer, 2014).

Q and I^2^ tests were used to test the heterogeneity of effect sizes. Another measure that shows heterogeneity and is easier to interpret for clinicians is the I^2^ value. This value ranges from 0% to 100%, indicating to what extent the total variability depends on the inter-study variability (heterogeneity). A value of 0% makes us think that the variability is due to sampling error or chance, while values approaching 100% suggest that the variability is largely due to the true heterogeneity between studies. As an arbitrary value, over 50% is interpreted as moderate, and above 75% as serious heterogeneity. Beg's and Egger's tests were used to test publication bias. In addition, Tau coefficient and p value greater than 0.05 indicates that there is no publication bias (Dinçer, 2014; Soleimani et al., 2020). Since the significance level was taken as 0.05 in the studies included, 0.05 was determined as the significance level of the statistical analyzes in this study. At the end of the test, it was found that the studies were heterogeneous (p < 0.05).

## Results

### Results of Studies Included in the Systematic Review

The study characteristics are displayed in Table 1. The included studies were published between 2020 and 2021 (Damirchi et al., 2020; Gokdemir et al., 2020; Guner et al., 2021; Kandemir, 2020; Mirhosseini et al., 2021; Rababa et al., 2021; Sanadgol et al., 2020; Shakil et al., 2021; Söğütlü & Göktaş, 2021). The total number of participants in the studies included in the meta-analysis is 4324. 2250 of the participants are women, 2071 are men, and 3 participants did not specify their gender. The ages of the individuals in the sample range between 15–64. Studies included in meta-analysis are designed in a descriptive way.

Guner et al. (2021): The study was conducted to examine the effect of loneliness experienced by the elderly during the COVID 19 epidemic on death anxiety. As a result of the study, a significant difference was found between death anxiety and loneliness in the elderly. In the study, the mean death anxiety score was found to be 8.54 ± 4.82. Considering the scores obtained from the scale, 5–9 points indicate a moderate death anxiety.

Söğütlü and Göktaş (2021): The aim of the study was to examine the health anxiety, death anxiety and physical symptoms caused by the pandemic in university students who were given a break from formal education and started to spend most of their days at home. As a result of the study, it was found that they have a moderate level of death anxiety.

Damirchi et al. (2020): The study investigated the role of self-talk in predicting death anxiety, obsessive-compulsive disorder, and coping strategies in the face of COVID-19. Data were collected by applying Self-Talk Scale (STS), Templer Death Anxiety Scale (DAS), Maudsley Obsessive-Compulsive Inventory (MOCI), Folkman and Lazarus Coping Strategies Inventory to 300 adults living in Ardebil, Iran. Death anxiety mean score was found to be 2.77 ± 1.8. In addition, significant negative relationships were found between self-talk and emotional coping style, death anxiety, and obsessive-compulsive disorder. According to the results of the regression analysis, it was determined that self-talk predicted problem-centered style, emotional coping style, death anxiety, and obsessive-compulsive disorder (Damirchi et al., 2020).

Gokdemir et al. (2020): The first aim of their study with 240 family physicians from eight countries was to examine family physicians' knowledge about COVID-19. The second aim was to evaluate their attitudes surrounding the current pandemic, stress and death anxiety. The data of the study were collected online using The Ten-Item Personality Inventory (TIPI), Satisfaction with life scale (SWLS), Perceived stress scale, Death anxiety scale: Templer Death Anxiety Scale (DAS) scales. Death anxiety mean score was found as 6.56 ± 3.01. 0 points of the scale indicate no death anxiety, 15 points indicate very high death anxiety, and a cut-off point of 6–7 indicates the symptoms of very high death anxiety. It is observed that the death anxiety of the participants is at a moderate level.

Mirhosseini et al. (2021): The study aimed to determine the death anxiety rate and covariates during the COVID-19 outbreak. As a result of the study, death anxiety was found to be 6.46 ± 3.33. Considering the minimum and maximum scores of the scale it can be said that the death anxiety rate is moderate.

Sanadgol et al. (2020): The study aimed to determine the effect of guided imagination on death anxiety in nurses working in the COVID-19 intensive care unit. The study, which was planned as a semi-experimental (pre-test-post-test), was carried out on 50 ICU nurses in a teaching hospital of Zabol Medical Sciences University in southeastern Iran. In the intervention group, the participants took imaginations with the guidance of lessons, brochures, booklets, and applications by using theory education. Participants in the control group did not receive any special intervention. In both groups, death anxiety was assessed by the investigator using the Templer DAS scale at the beginning of the study and one month after the intervention. As a result of the study, the death anxiety rate of the intervention group was found to be 53.28 ± 7.54. Death anxiety of the control group was found to be 50.84 ± 7.63. Considering the minimum and maximum values of the scale (15–75) it is seen that death anxiety levels are quite high. At the end of the study, the mean death anxiety score of imagination with the guidance of 1 month decreased significantly from 53.28 to 43.48 (p = 0.01). There was no significant difference in the control group before and after the intervention (p > 0.05).

Shakil et al. (2021**):** In the study, death anxiety and psychological distress were examined in the context of working status during the COVID-19 pandemic process. Demographic information, Psychological Distress Scale-K10, Death Anxiety Scale scales were applied to a sample group of 478 people from the general population to collect data. The mean score of death anxiety was found to be 27.79 (6.05). Considering that the min/max scores of the scale are 15–75 and 15–35 indicates low death anxiety. By showing the strong relationship between psychological distress and death anxiety for the non-working participants, it points to a meaningful moderator role of the work situation. Therefore, psychological distress is associated with more pronounced death anxiety in non-working people than in workers. This study highlights the importance of working as a potential anxiety-buffering factor regarding thoughts about death due to psychological distress.

Kandemir (2020): In the study, the relationship between the demographic characteristics of individuals and their level of religiosity and death anxiety during the COVID-19 pandemic process. Personal Information Form, Religious Orientation Self-Recognition Inventory and Death Anxiety Scale were used for individuals consisting of 660 people. The minimum and maximum values of the scale are 0 and 100. The higher the score obtained from the scale indicates the higher the death anxiety. The death anxiety ratio of the participants was found to be 48.0 ± 15.3. According to this result, it is seen that the death anxiety of the participants is at a moderate level. It revealed the existence of statistically significant relationships between the variables of gender, age and marital status of the sample and the variables of religiosity and death anxiety. It was determined that the socio-economic level variable did not have a significant relationship with neither religiousness nor death anxiety variable.

Rababa et al. (2021): The study was conducted to examine the relationship of death anxiety to religious coping and spiritual well-being among 248 older adults living in the community during the COVID-19 pandemic. Arabic Religious Coping Scale, Arabic Version of Spiritual Wellbeing Scale and Arabic Death Anxiety Scale were used to measure religious coping, spiritual well-being, and death anxiety, respectively. The death anxiety mean score of the participants was found to be 68.76 ± 19.2. The lowest score to be obtained from the scale is 20 and the highest score is 100. Most of the older adults who participated were found to have low levels of religious coping and spiritual well-being, and high levels of death anxiety. Moreover, compared to older male adults, female older adults have been found to have higher levels of religious coping and lower levels of death anxiety. It was also found that married older adults have higher levels of death anxiety compared to widowed older adults. After controlling for sociodemographic characteristics, religious coping and mental well-being were found to be important predictors of death anxiety in older adults.

### Results of Studies Included in the Meta-Analysis

It is the examination of the associated mean values ​​for a variable. Therefore, the effect size includes relations with one variable. Table 2 gives the effect sizes, standard deviations, variables, Z and p values ​​of the studies included in the study. The averages of the scales used in all of these studies represent the effect size. Here, the effect size for each study is represented by a square. Both the direction and the magnitude of the impact are expressed in terms of the location of this square. In this study, the effect size was calculated on basis of averages and the squares were distributed between 8.540 and 2.770 values. Accordingly, the study with the highest effect size is Guner et al. (2021) (8.540), while the lowest one is Damirchi et al. (2020) (2.770). When looking at the general effect size, it is seen that the effect size is 6.087. According to these results, death anxiety is moderate.

**Table 2.** About the Death Anxiety Outcomes.

Author, Year	Country	Sample size		Death Anxiety Skoru	Meta-analytic effect size					Heterogeneity test			
					Mean	Standard error	95% CI	Z value	P value	Q	Df (Q)	P	I^2^
1. Damirchi et al. (2020)	Iran	300	18–54 adults	TDAS: 2.77 ± 1.8	6.087	0.048	5.992 6.181	126.190	0.0000	1383.085	4	0.000	99.711
2. Gokdemir et al. (2020)	Türkiye	240	Family Physicians	TDAS: 6.56 ± 3.01									
3. Guner et al. (2021)	Türkiye	354	Elderly	TDAS:8.54 ± 4.82									
4. Söğütlü & Göktaş (2021)	Türkiye	987	18–26 Age	TDAS: 7.25 ± 2.06									
5. Mirhosseini et al. (2021)	Iran	1215	General Population	TDAS: 6.46 ± 3.33									
6. Shakil et al. (2021)	Pakistan	468	General Population	TDAS: 27.79 ± 6.05	29.336	0.271	28.805 29.866	108.404	0.000	481.248	2	0.000	99.584
7. Sanadgol et al. (2020)	Iran	25	General Population	TDAS: 53.28 ± 7.54									
8. Sanadgol et al. (2020)	Iran	25	General Population	TDAS: 50.84 ± 7.63									
9. Kandemir (2020)	Türkiye	319	Female	ÖKÖ: 52.5	51.25	0.51	50.25 52.26	100.37	0.000	320.408	2	0.000	99.376
10. Kandemir (2020)	Türkiye	341	Male	ÖKÖ: 43.2									
11. Rababa et al. (2021)	UAE	248	Older Adults	ASDA:68.76 ± 19.2									

**Figure 2.**
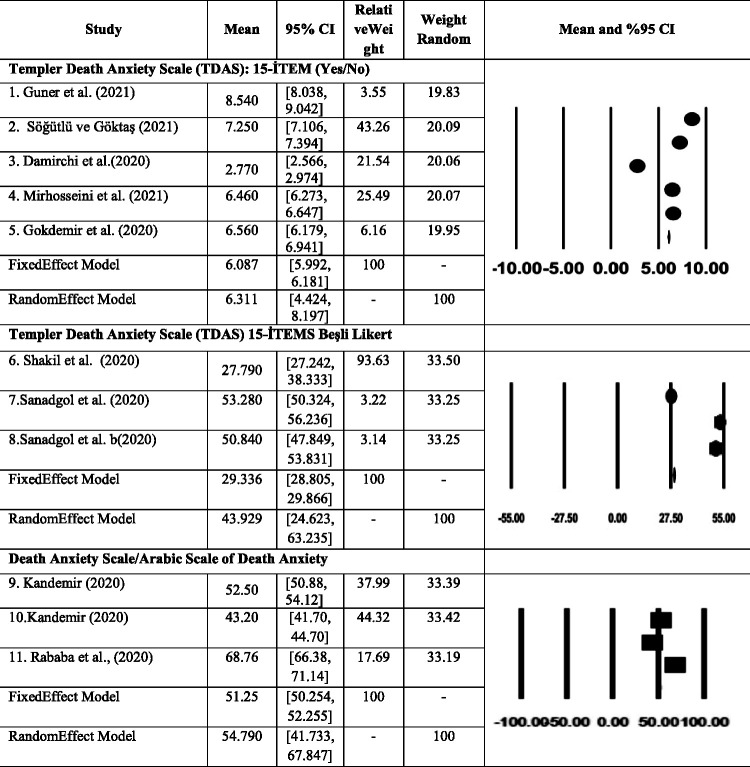
The Forest Plot About the Death Anxiety Outcomes.

In some meta-analyzes, it is seen that heterogeneity is taken as a stand-alone criterion. It is seen that the random effects model is preferred if the heterogeneity is significant, and the fixed effect model is preferred if it is not. However, the general opinion is that heterogeneity should not be taken as a ‘stand-alone' criterion in model selection. It should not be forgotten that heterogeneity can be found false-negative, especially in meta-analyzes involving a small number of studies. Another measure that shows heterogeneity and is easier to interpret for clinicians is the I^2^ value. Although I^2^ is easy to interpret in this way, it has the limitation of being a relative measure and not showing absolute heterogeneity. Q value or p value can also be taken into account to determine the homogeneity (or heterogeneity) of the study (Dinçer, 2014). As a result of this study, in which the death anxiety rate was examined using the TDAS (15 items; yes/no form) scale, the heterogeneity test had a Q value of 1383.085 and a p value of less than 0.05 (p = 0.000 < 0.05). It seems to be in structure. Estimated mean of death anxiety (TDAS) is 6.087 (CI95% = [5.992, 6.181]; I^2^ = 99.71%; Z = 126.19, p < 0.001; Q = 1383.085) (Table 2). The forest plot is given in Figure 2.

Table 2 TDAS (15-item five-point Likert) means are distributed between 27,790 and 53,280 values. Accordingly, the study with the highest effect size is Sanadgol et al., 2020 (53,280), while the lowest one is Shakil et al., 2021 (27,790). When looking at the general effect size, it is seen that the effect size is 29,336. According to these results, death anxiety is at a low level. As a result of this study in which the death anxiety rate was examined using the TDAS (15-item five-point Likert) scale, it was observed that the studies included in the analysis had a heterogeneous structure because the Q value of the heterogeneity test was 295.859 and the p value was less than 0.05 (p = 0.000 < 0.05). Estimated mean of death anxiety (TDAS) 29.336 (CI95% = [28.805, 29.866]; I^2^ = 99.58%; Z = 108.404, p < 0.001; Q = 481.248) (Table 2). The forest plot is given in Figure 2.

The effect size of the studies in which the death anxiety scale was used in Figure 2 was calculated based on the averages and the squares were distributed between the values of 43.20 and 68.76. Accordingly, the study with the highest effect size is Rababa et al. (2021) (68.76), while the lowest one is Kandemir (2020) (43.26). When looking at the general effect size, it is seen that the effect size is 51.25. According to these results, death anxiety is moderate. As a result of this study, in which the death anxiety rate was examined using the Death Anxiety Scale scale, it was seen that the studies included in the analysis had a heterogeneous structure, since the Q value of the heterogeneity test was found to be 295.859 and the p value was less than 0.05 (p = 0.000 < 0.05). RDAS estimate mean 51.25 (CI95% = [50.25, 52.26]; I2 = 99.376%; Z = 100.37, p < 0.001; Q = 320.408). The forest plot is given in Figure 2. It was evaluated according to Beg's and Egger's tests and funnel plot. All tests confirmed the non-significant publication bias (p > 0.05).

## Discussion

The purpose of reviewing the entire evidence-base is to provide access guide for health professionals, encourage the uptake of evidence-based interventions, to confer better outcomes for patients. The purpose of this article is to review the death anxiety during the COVID-19 pandemic process in different populations, and to systematically define the characteristics and associated variables of people with high death anxiety. In this direction, the current study was conducted to analyze the studies conducted on evidence-based research results on the examination of death anxiety in the COVID-19 pandemic process. Although there are many descriptive studies on the effect of death anxiety in the COVID-19 pandemic process, there is no meta-analysis or systematic review study. This study is the first study conducted in terms of evidence-based practices on this subject. Five of the studies included in the meta-analysis were conducted in the general population, two in elderly adults and one in intensive care nurses and one in family physicians. As a result of the study, although death anxiety levels vary, considering the effect sizes of the studies, it is seen that death anxiety is high in studies conducted with intensive care nurses, family physicians and elderly adults. Similarly, when we look at previous epidemics, it is seen that death anxiety rates are high. For example, as a result of a study conducted in Mexico, the incidence of death anxiety in the influenza A/H1N1 epidemic was reported to be 17% (Elizarrarás-Rivas et al., 2010). It can be said that the reason for this is that there is a vital threat like a pandemic, and the emotions and thoughts about death anxiety become apparent. When the literature is examined, as a result of the study of Turhan(2021), it has been determined that the COVID-19 epidemic has triggered death anxiety since December 2019 (Turhan, 2021). A study was conducted by Çağlar and Kaçer (2021) to compare death anxiety levels of patients with COVID-19 pneumonia, MI patients and healthy participants. As a result of this study, it was found that death anxiety levels of patients with COVID-19 pneumonia were as high as patients with MI. The reason for this may be the high mortality rate worldwide, quarantine measures, individuals getting away from their routine lifestyles, and the absence of a specific treatment for the disease (Çağlar & Kaçer, 2021). Review studies suggest that death anxiety in the COVID-19 pandemic process is experienced at a higher level in groups considered as a risk group compared to regular populations.

It is known that death anxiety is affected by many factors. These include gender, marital status, age, belief, frequency of encountering death, near-death experiences. However, in general, individuals can feel death anxiety intensely when they feel their own lives are indirectly in danger (Ceylan, 2018). As a matter of fact, studies provide evidence that death anxiety is affected by changes such as gender, age and marital status (Kandemir, 2020; Özcan et al., 2019). For example, as a result of the study conducted by Saleem and Saleem (2020), one of this evidence is that men experience a lower level of death anxiety (Saleem & Saleem, 2020).

Although there is no systematic review reporting death anxiety seen during the COVID-19 process, some studies have addressed anxiety or death anxiety in races and professions (Curșeu et al., 2021; Damirchi et al., 2020; Kavaklı et al., 2020; Lázaro-Pérez et al., 2020; Pappa et al., 2020; Pradhan et al., 2020; Salari et al., 2020; Santabárbara et al., 2021). In particular, the death anxiety rates of healthcare workers who are at the center of the fight against the COVID-19 pandemic are high (Cudris-Torres et al., 2020; Indacochea et al., 2021; Sanadgol et al., 2020). It is noteworthy that death anxiety is high for doctors treating COVID-19, especially in environments without protective equipment (Indacochea et al., 2021).

It has been stated that old age has been an important risk factor since the beginning of the pandemic (Wang et al., 2020). The high prevalence of death anxiety among the elderly is due to many physical problems, chronic illnesses, movement disorders, physical disabilities, and dependence on others. Retirement and subsequent loneliness can also contribute to death anxiety in the elderly (Birgit et al., 2018; Menzies & Menzies, 2020; Ring et al., 2020). It is also known that during this period, people's lives are more prone to anxiety due to reduced activity and mobility, loss of friends, reduced financial and physical independence, and chronic diseases (Mohammadpour et al., 2018; Mokhtari et al., 2020). Confirmed or suspected cases of COVID-19 infection can contribute to stress and anxiety, especially among the elderly (Li et al., 2020; Meng et al., 2020). In addition, the strong association between age and physical morbidity due to COVID-19 can cause higher health concerns among older adults. These health and health concerns can also cause an increase in death anxiety (Ring et al., 2020). In a study conducted to determine mortality rates from COVID-19 over the age of 65, it was found that individuals aged 65 and over had strikingly higher COVID-19 mortality rates compared to young individuals (Yanez et al., 2020). Therefore, we can say that the reason why death anxiety rates are higher in the elderly is that deaths due to COVID-19 are higher in the elderly.

Death anxiety appears to emerge as an abnormal experience when people face threats to mortality for reasons such as experiencing or fear of COVID-19 (Menzies & Menzies, 2020; Menzies et al., 2020). The results of this study provide evidence that the COVID-19 pandemic causes more death anxiety, mostly in healthcare workers, women and the elderly compared to younger people. When it comes to family and children, it can be thought that women are affected more psychologically (Kavaklı et al., 2020; Zhang et al., 2020). Similarly, in the study conducted by Kavaklı et al. (2020), it was proven that female participants perceive higher death anxiety than male participants during the pandemic process (Kavaklı et al., 2020). Death anxiety can lead to situations such as loneliness, depression, and increasing the frequency of hospital admissions. This study revealed that only studies with participants during the COVID-19 pandemic process reported higher death anxiety and suggested that women may experience more death anxiety. Other research has confirmed that both death anxiety and psychological distress are greater among women. Men think about death more often than women but have lesser negative feelings and less anxiety about death. The findings of this study revealed the high level of death anxiety in the COVID-19 process, based on the relevant literature, and drew attention to which groups were most affected by this anxiety. The link between different races and death anxiety is largely unknown, possible factors including religiosity and cultural norms must be considered. It is recommended to conduct studies examining death anxiety and these factors.

The results of this study should be handled carefully. Although there are very few research results, the studies compiled in the study and the results obtained from them show the death anxiety problem, and it can be regarded as a good sign for studies addressing this problem to be conducted and disseminated. From this point on, it seems important to increase the number of therapeutic studies to be carried out with individuals experiencing death anxiety. It should be kept in mind that institutions serving individuals with death anxiety or potential to survive will guide them in organizing and implementing effective programs.

The COVID-19 pandemic, with its increasing case and death rates, also causes various effects on human psychology. Therefore, it is recommended to evaluate the COVID-19 pandemic, which can be perceived as a threat to human existence, in terms of death anxiety in risky groups (elderly/women). It is recommended to provide support services that individuals with high death anxiety can access online when they need it, taking into account the pandemic conditions. In this context, tele-psychiatry services can be expanded to reach more people.

## Conclusion

The evidence generated through a systematic review can provide the mental health professionals with greater confidence in decision making at the moment of practice and optimize the benefits to people with high death anxiety, serving as a tool to assist managers in implementation of new strategies in favor of the mental health. The findings of this study suggest that death anxiety is relatively high during the COVID-19 pandemic process and is affected by socio-demographic factors such as fear of COVID-19, gender, and occupation. During the COVID-19 pandemic, it was determined that mostly healthcare workers, women and the elderly have more death anxiety than younger people. The valuable information of the present study can be used as a starting point for preventing death anxiety and for appropriate interventions in individuals at risk. It is recommended to conduct randomized controlled studies addressing death anxiety during the COVID-19 pandemic process.

Factors associated with death anxiety were determined as death of a family member from COVID-19, religiosity and cultural norms, perceived level of stress, attitude towards COVID-19, subjective proximity to death, coping strategies, history of COVID-19 contact, mental illness, alcohol consumption, loneliness, perceived risk and strategies for coping with stress.

When the literature on the pandemic period was examined, it was found that most of the studies were planned as cross-sectional and relationship seekers, but randomized controlled experimental studies were lacking. Considering that Randomized Controlled studies constitute second-level evidence in making decisions about implementation, it is important to plan studies to eliminate this deficiency.

### Limitations and Strengths

This study is the first to systematically review the death anxiety averages. The strength of this work is the meticulous research process of academic databases. In addition, the studies were examined in terms of quality and heterogeneity. To fill the gaps identified in the literature, prospective research can be done and designed with stronger data collection methods.
